# Supplementary material for: Isolating Small Extracellular Vesicles from Small Volumes of Blood Plasma using size exclusion chromatography and density gradient ultracentrifugation: A Comparative Study
Source: bioRxiv. 2025 Jan 22:2023.10.30.564707. Originally published 2023 Nov 1. Preprint. [Version 2] doi: 10.1101/2023.10.30.564707 (PMC10634961; doi:10.1101/2023.10.30.564707)
Supplement: Supplement 1 [file NIHPP2023.10.30.564707v2-supplement-1.pdf]

bioRxiv preprint doi: https://doi.org/10.1101/2023.10.30.564707; this version posted January 22, 2025. The copyright holder for this preprint (which was not certified by peer review) is the author/funder, who has granted bioRxiv a license to display the preprint in perpetuity. It is made available under aCC-BY 4.0 International license.

Supplementary Figure Data:

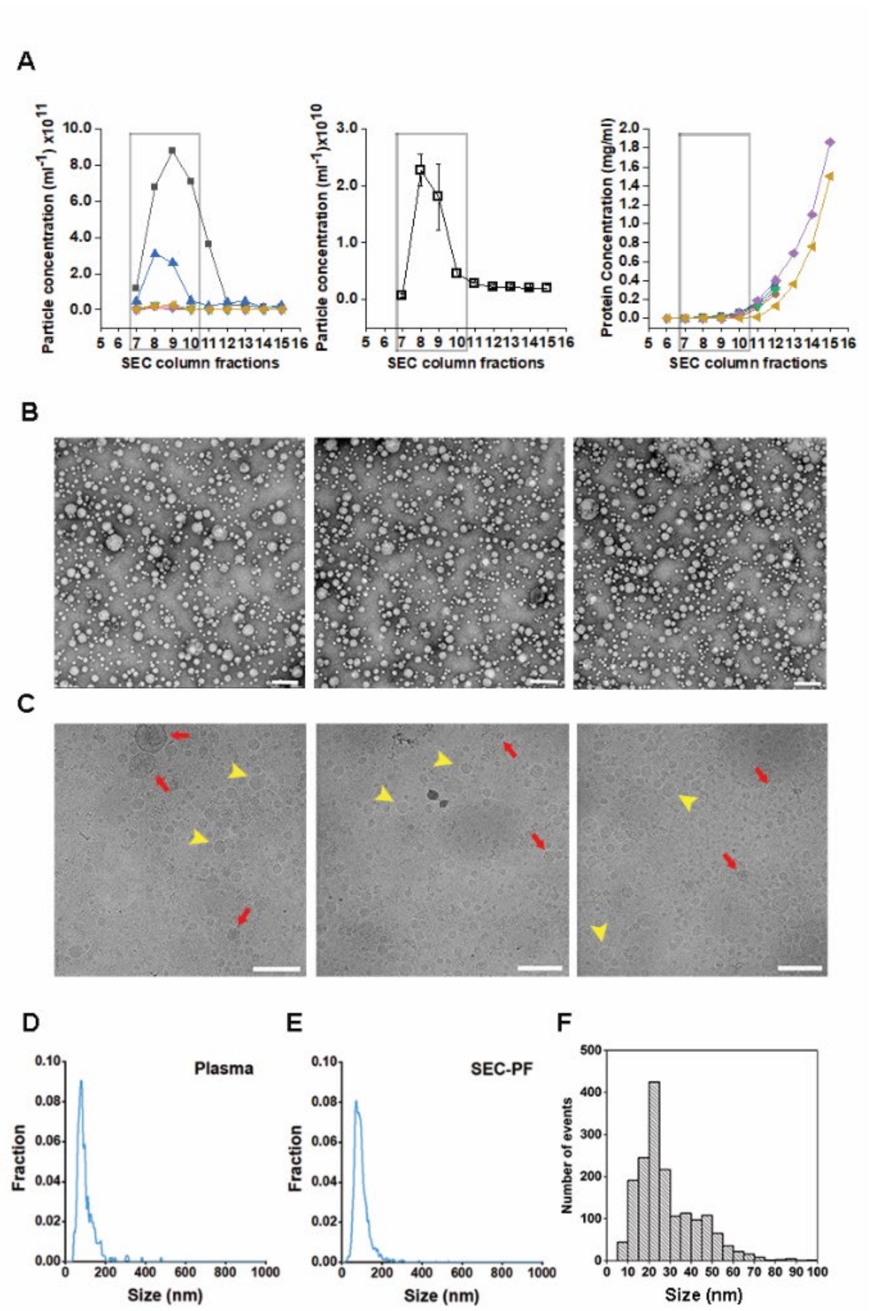

Supplementary Figure 1: SEC elution profiles, EM images of the PF and particle size distributions. (A) SEC elution profiles according to particle concentration (by NTA) of 6 different plasma sources (left) and 4 repeats of the same plasma source (middle). Protein concentrations (by BCA, right) in corresponding to the elution profiles of the 6 different plasma sources shown on the left. 500 μl of plasma was loaded onto the SEC column and 10 fractions of 500 μl each were collected and analyzed. Fraction 7 to 10 were pooled to constitute the SEC-PF because of their high particle concentrations and low protein concentrations. (B) Representative TEM images of the PF. (C) Cryo-EM images of the SEC-PF. (D), (E) Typical particle size distributions of a plasma and SEC-PF measured by NTA. (F) A histogram of particle diameters obtained from the TEM images shown in (B).

bioRxiv preprint doi: https://doi.org/10.1101/2023.10.30.564707; this version posted January 22, 2025. The copyright holder for this preprint (which was not certified by peer review) is the author/funder, who has granted bioRxiv a license to display the preprint in perpetuity. It is made available under aCC-BY 4.0 International license.

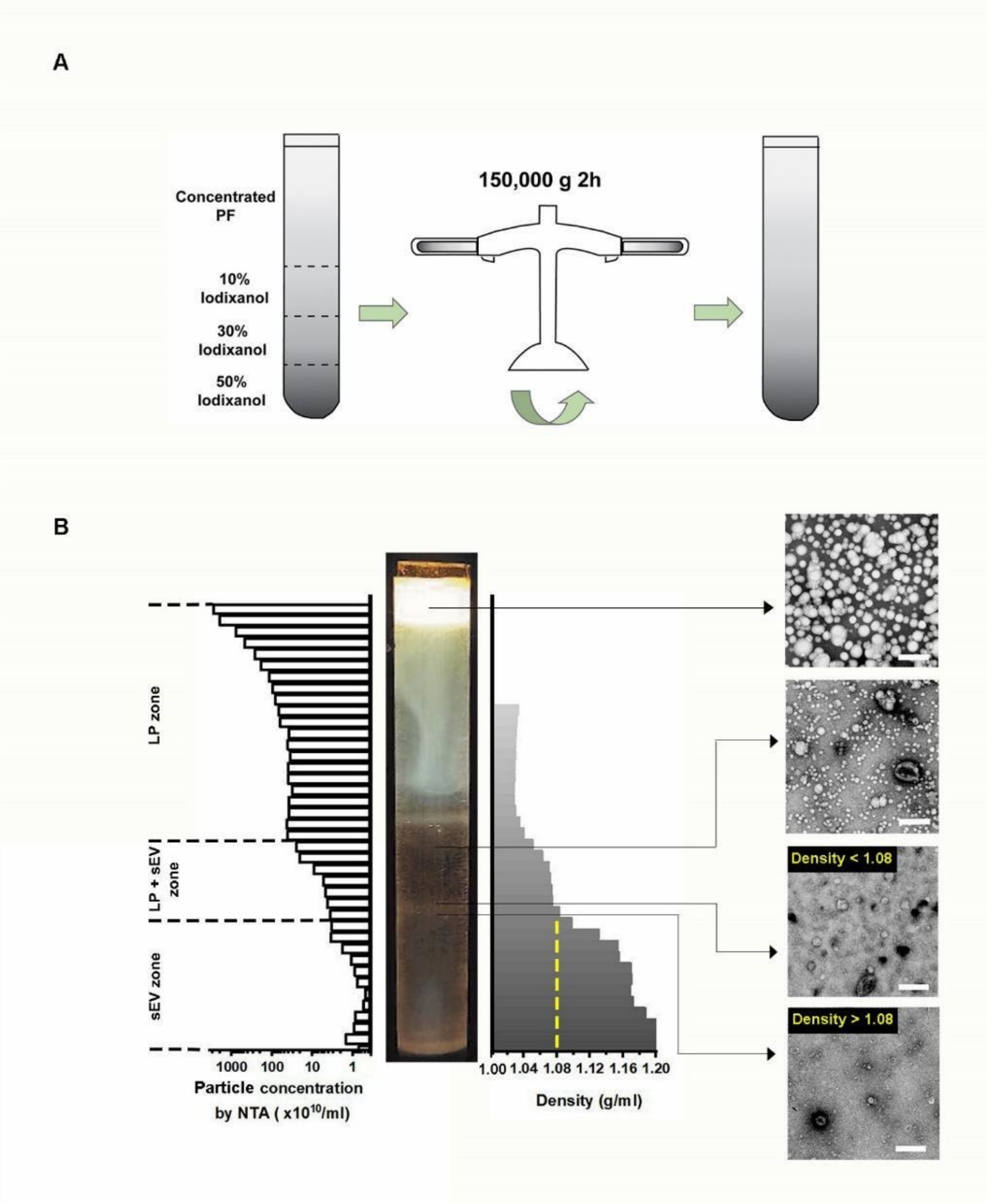

Supplementary Figure 2: Distribution of sEVs and lipoproteins within the density gradient after DGUC. (A) SEC-PFs (6 *ml*) were placed on top of a density gradient cushion constructed with 2 *ml* 10%, 2 *ml* 30%, and 2 *ml* 50% iodixanol solutions and centrifuged at 150,000 x *g* for 2 *h* at 4 ℃. (B) After DGUC, the tube was fractionated into 42 fractions, which were each examined for their densities, particle concentrations (by NTA) and presence of sEVs and lipoproteins (by TEM). The dominance of lipoproteins was evident in fractions of density <1.05 *g/ml,* which is designated as LP zone. sEVs started to appear when density exceeded 1.05 *g/ml* but a significant level of lipoproteins was present until the density of 1.08 *g/ml*. Therefore, the density region of 1.05~1.08 *g/ml* was designated as

bioRxiv preprint doi: https://doi.org/10.1101/2023.10.30.564707; this version posted January 22, 2025. The copyright holder for this preprint (which was not certified by peer review) is the author/funder, who has granted bioRxiv a license to display the preprint in perpetuity. It is made available under aCC-BY 4.0 International license.

LP+sEV zone. Beyond 1.08 *g/ml*, lipoproteins diminished drastically and the density region of >1.08 *g/ml* was designated as the sEV zone. All scale bars represent 200 *nm*.

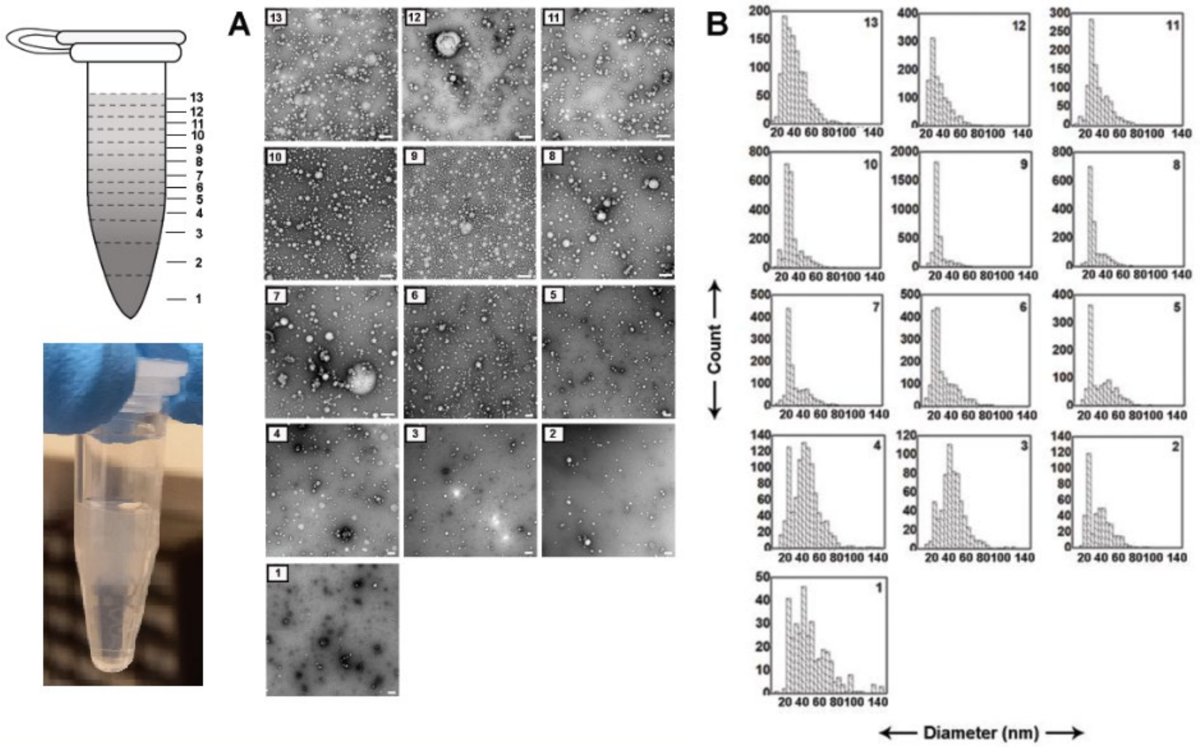

Supplementary Figure 3: TEM images and size distributions of the 13 fractions collected from the 1.5 *ml* tube following SEC-DGUC. (A) Representative TEM images of each of the 13 fractions. SEC-DGUC-1 clearly showed minimum presence of lipoprotein (high contrast lighter color particles) compared to other fractions. Moreover, the presence of sEVs (low contrast and cup-shaped particles) is evident in SEC-DGUC-1. A typical image of the 1.5 *ml* tube after the DGUC step is shown at the left (bottom picture). The whitish layer at the top, gradually becoming clearer toward the bottom of the tube, corresponds well with the TEM observations. (B) Particle size distributions measured according to the TEM images of the 13 fractions.

bioRxiv preprint doi: https://doi.org/10.1101/2023.10.30.564707; this version posted January 22, 2025. The copyright holder for this preprint (which was not certified by peer review) is the author/funder, who has granted bioRxiv a license to display the preprint in perpetuity. It is made available under aCC-BY 4.0 International license.

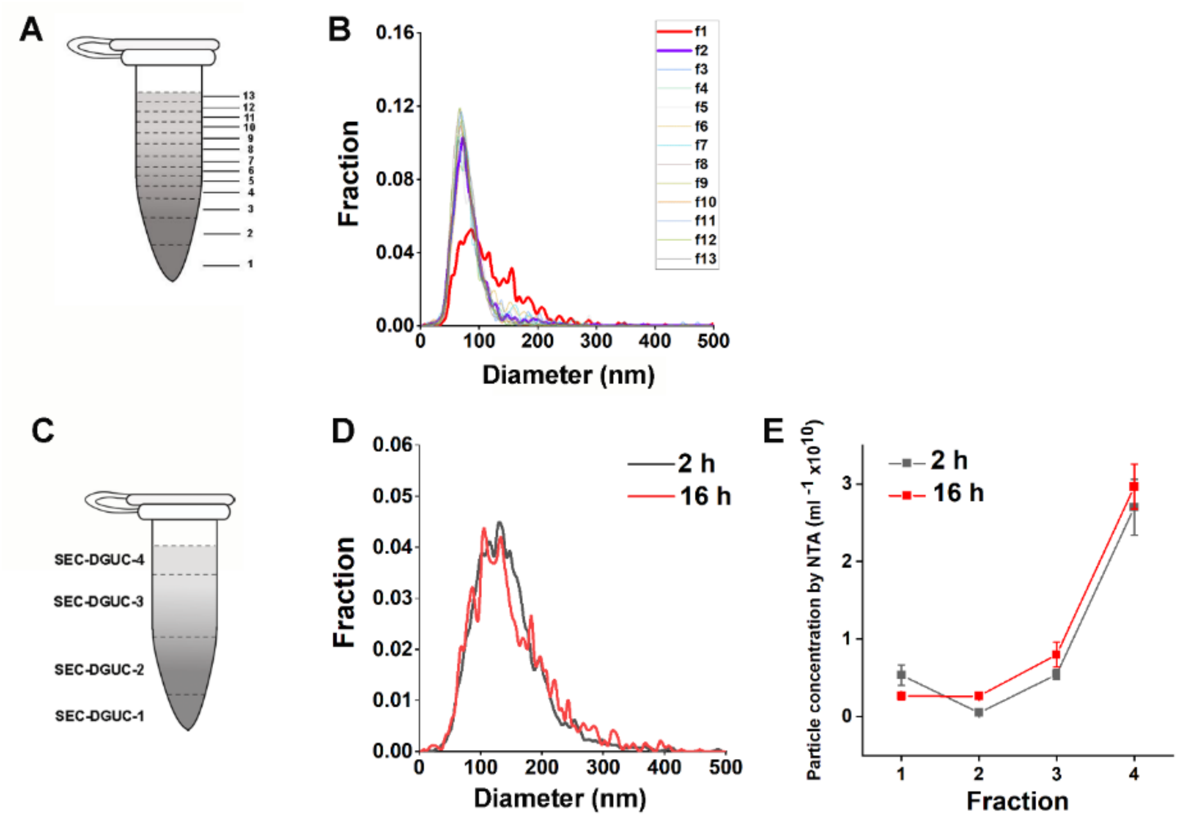

Supplementary Figure 4: (A) The 1.5 *ml* tube was fractionated into 13 fractions. (B) Size distributions (by NTA) of the 13 fractions collected from the 1.5 *ml* tube following SEC-DGUC protocol. The particle size distribution in SEC-DGUC-1 was distinct from the rest of the fractions. Even in SEC-DGUC-2 (highlighted in purple), the size distribution follows fractions 3~13, implying the dominance of lipoproteins in these fractions. (C) The 1.5 *ml* tube was fractionated into 4 fractions in order to examine if 2 *h* spinning time was sufficient to isolate sEV from lipoproteins in the 1.5 *ml* tube format DGUC. (D) The resulting size distributions of SEC-DGUC-1s (by NTA) were highly similar for 2 *h* and 16 *h* spinning time. (E) The particle concentrations of the four individual fractions along the 1.5 *ml* tube closely resembled each other for 2 *h* and 16 *h*, implying that there were no significant loss of sEV for 2 *h* spinning time in comparison to 16 *h*.

bioRxiv preprint doi: https://doi.org/10.1101/2023.10.30.564707; this version posted January 22, 2025. The copyright holder for this preprint (which was not certified by peer review) is the author/funder, who has granted bioRxiv a license to display the preprint in perpetuity. It is made available under aCC-BY 4.0 International license.

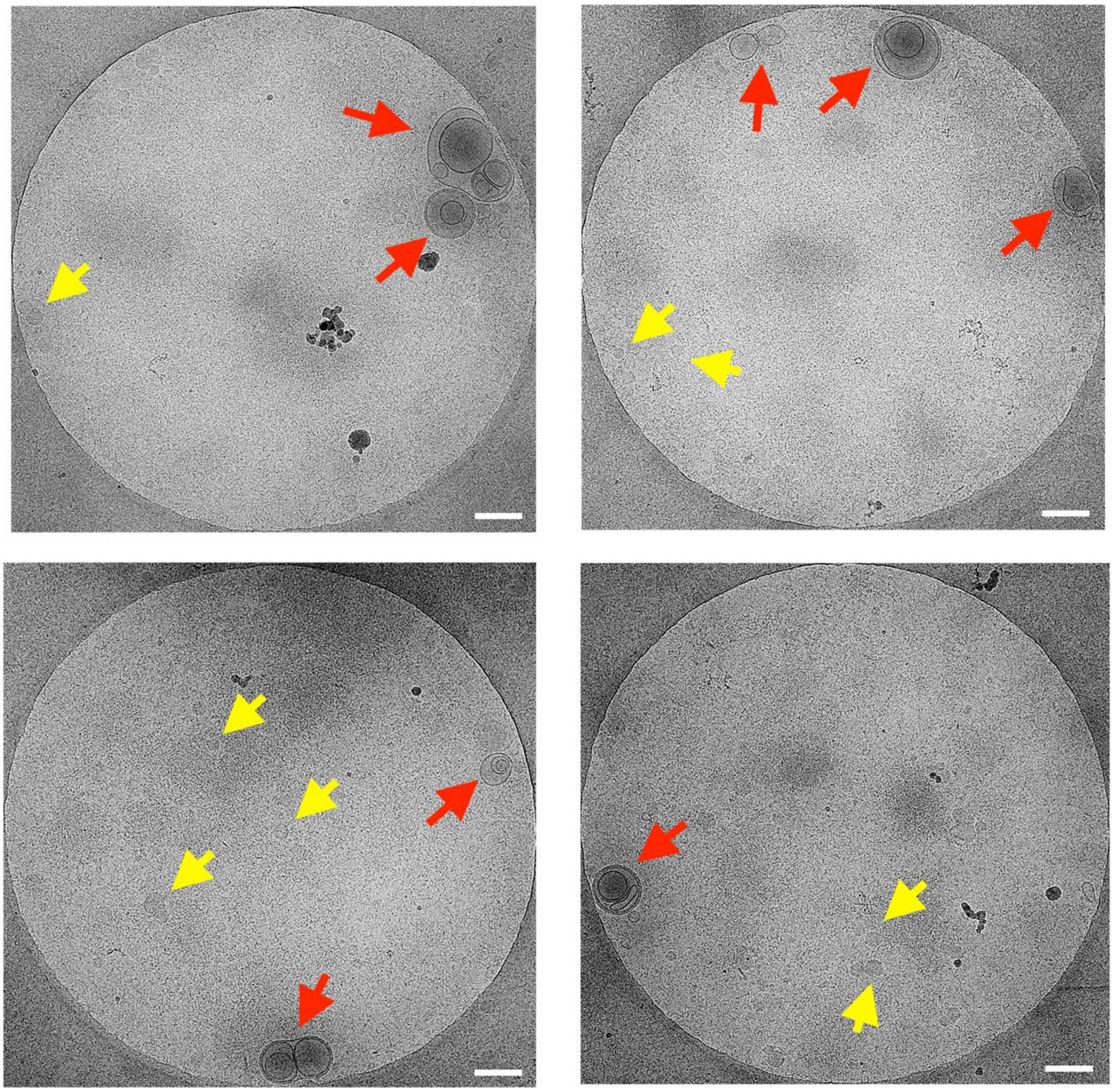

Supplementary Figure 5: Additional Cryo-EM images of SEC-DGUC-1. Red arrows represent sEVs and yellow arrows represent typical lipoproteins. The SEC-DGUC-1 was obtained from non-fasting plasma collected in EDTA tubes. All scale bars represent 200 *nm*.

bioRxiv preprint doi: https://doi.org/10.1101/2023.10.30.564707; this version posted January 22, 2025. The copyright holder for this preprint (which was not certified by peer review) is the author/funder, who has granted bioRxiv a license to display the preprint in perpetuity. It is made available under aCC-BY 4.0 International license.

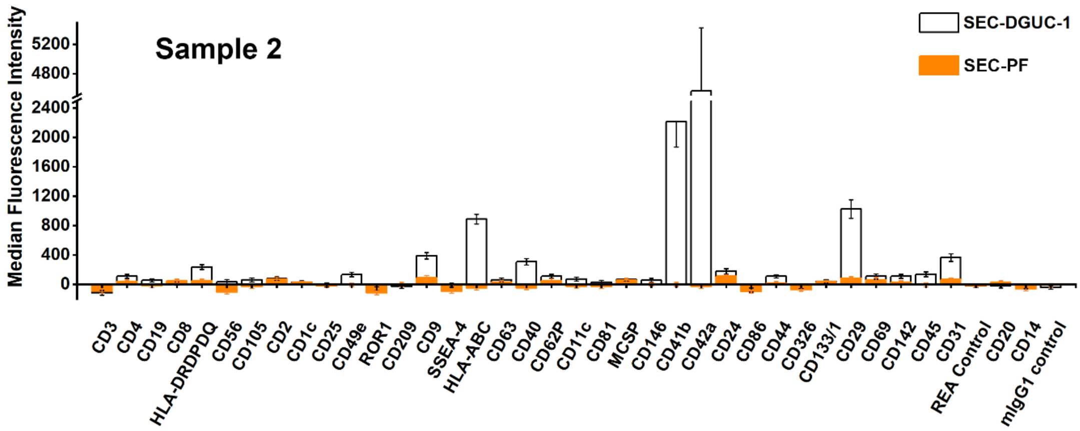

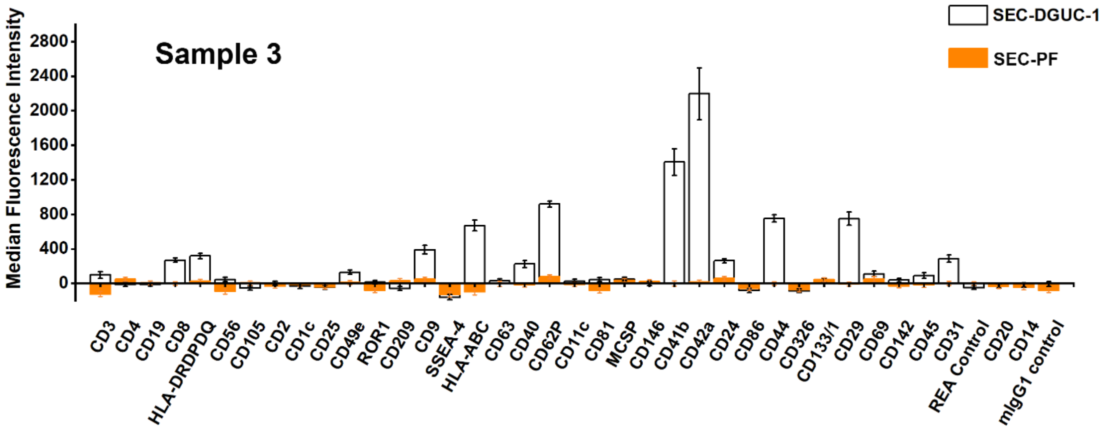

Supplementary Figure 6: Flow cytometry data of SEC-PF vs. SEC-DGUC-1 obtained from two plasma sources using MACSPlex exosome kit. Data from two plasma sources demonstrated a similar trend of much stronger signals of SEC-DGUC-1 compared to SEC-PF in flow cytometry using MACSPlex exosome kit. Data are Median ± SEM.

bioRxiv preprint doi: https://doi.org/10.1101/2023.10.30.564707; this version posted January 22, 2025. The copyright holder for this preprint (which was not certified by peer review) is the author/funder, who has granted bioRxiv a license to display the preprint in perpetuity. It is made available under aCC-BY 4.0 International license.

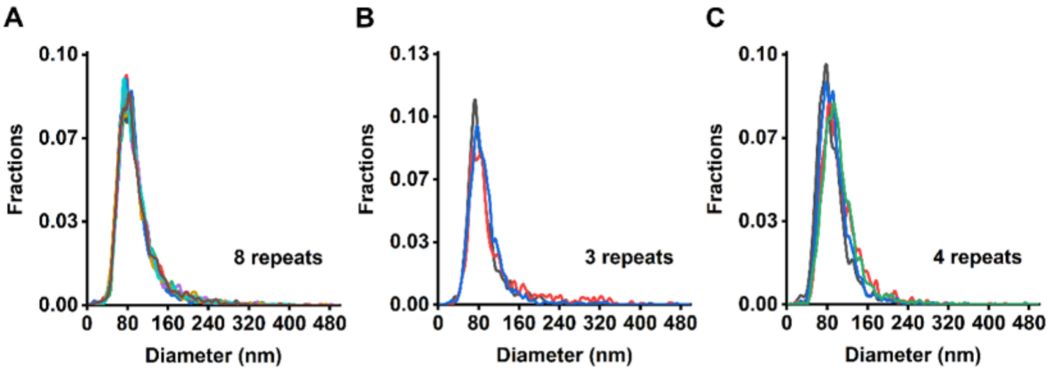

Figure number	Average vesicle concentration	Standard Deviation	SD/average ratio	Number of measurements
A	$1.6x10^{12}$	$4.2x10^{11}$	26%	8
B	$3.1x10^{11}$	$9.3x10^{10}$	30%	3
C	$2.2x10^{11}$	$1.2x10^{11}$	55%	4
	$8.6x10^{10}$	$2.9x10^{10}$	33%	3
	$4.7x10^{11}$	$2.8x10^{10}$	6%	3
	$9.6x10^{9}$	$7.0x10^{8}$	7%	3

Supplementary Figure 7: Repeatability of SEC. (A-C) Particles size distributions of SEC-PF measured by NTA. (A), (B) and (C) correspond to the first, second and third rows of the table, respectively. The highly overlapped size distributions allude to the consistency in the particles eluted from SEC columns, even though the number of particles eluted varied up to 55%. The table lists six experiments with various numbers of repeats of SEC. The average particle concentrations, standard deviation and CV (SD/average ratio) are listed together with the number of measurements made.

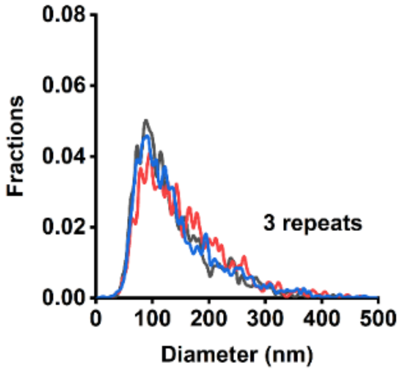

Average vesicle concentration	Standard Deviation	SD/average ratio	Number of measurements
$3.9x10^{9}$	$9.3x10^{8}$	24%	4
$2.0x10^{10}$	$5.0x10^{9}$	25%	3

Supplementary Figure 8: Repeatability of SEC-DGUC protocol. NTA measurements of two experiments to test repeatability after SEC-DGUC. The figure displays the size distributions measured by NTA of the experiment listed in the second row of the table. The size distributions of the experiment

bioRxiv preprint doi: https://doi.org/10.1101/2023.10.30.564707; this version posted January 22, 2025. The copyright holder for this preprint (which was not certified by peer review) is the author/funder, who has granted bioRxiv a license to display the preprint in perpetuity. It is made available under aCC-BY 4.0 International license.

listed in the first row of the table were presented in main Figure 9. The table lists the average particle concentrations of SEC-DGUC-1 and the standard deviation, CV (SD/average ratio) together with the number of measurements made.

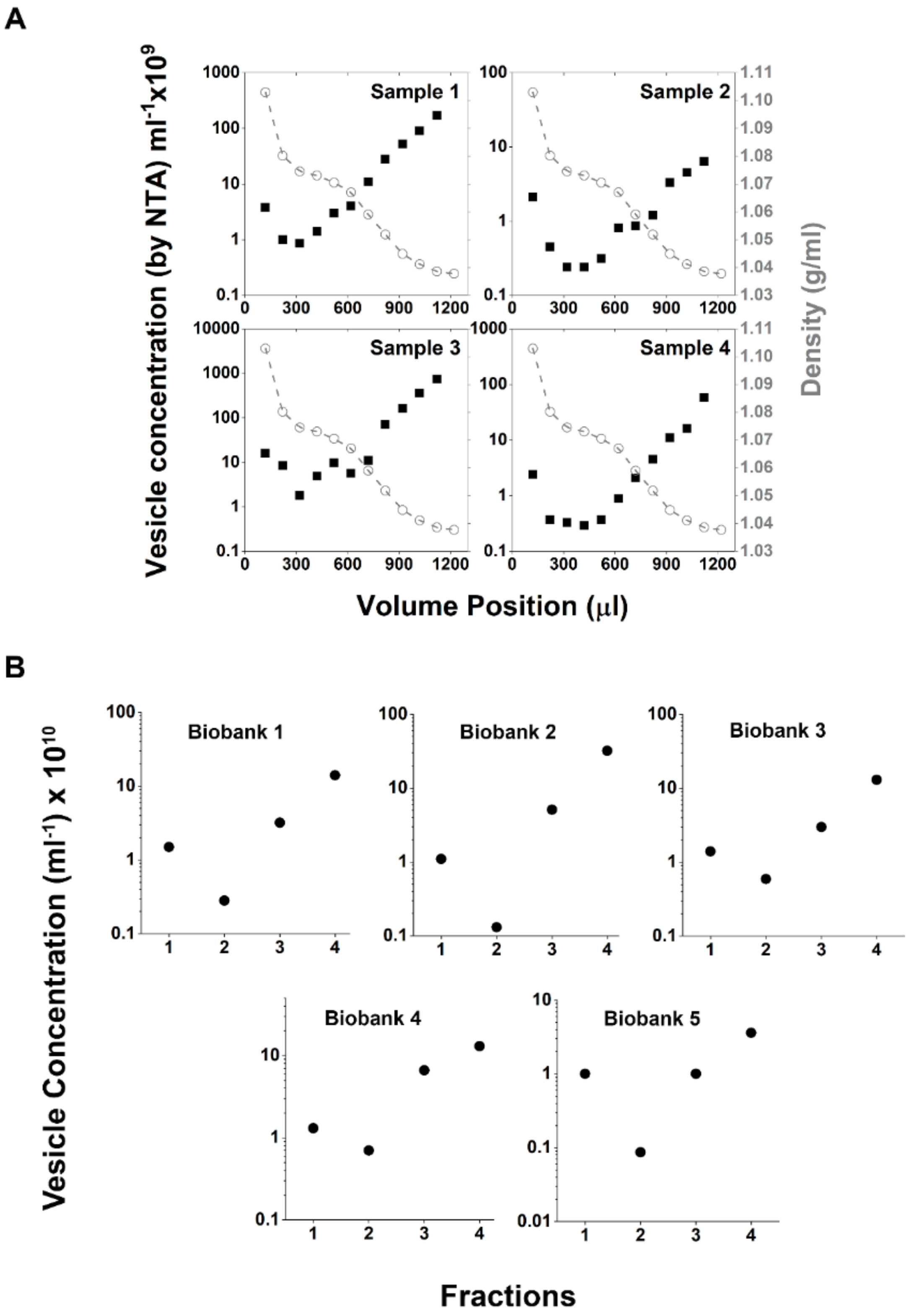

bioRxiv preprint doi: https://doi.org/10.1101/2023.10.30.564707; this version posted January 22, 2025. The copyright holder for this preprint (which was not certified by peer review) is the author/funder, who has granted bioRxiv a license to display the preprint in perpetuity. It is made available under aCC-BY 4.0 International license.

Supplementary Figure 9: Reliability of particle concentration profiles along the 1.5 *ml* tube (by NTA). (A) Detailed particle concentration profiles (13 fractions) of four different samples along with the calibrated density profile shown in Figure 2. (B) Five particle concentration profiles (4 fractions shown in Figure 9B) in SEC-DGUC-1 corresponding to the biobank plasma samples (EDTA tubes) shown in Figure 11. Note that the data from (A) and (B) are from different plasma sources.

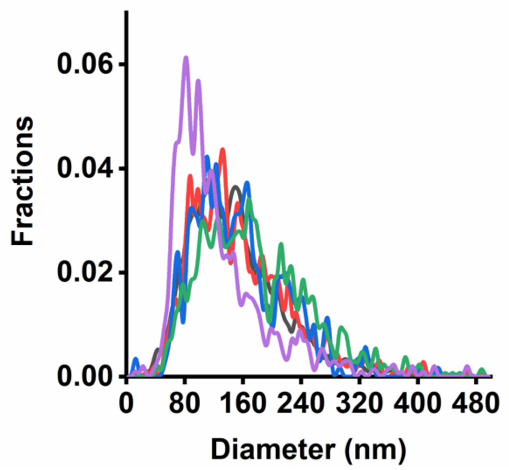

Supplementary Figure 10: Size distributions (by NTA) of SEC-DGUC-1 obtained from 5 fasting plasma corresponding to Figure 11. The particle size distributions did not overlap, reflecting the variation of sEV population among different plasma sources.
